# Persistent ER Stress Induces the Spliced Leader RNA Silencing Pathway (SLS), Leading to Programmed Cell Death in *Trypanosoma brucei*

**DOI:** 10.1371/journal.ppat.1000731

**Published:** 2010-01-22

**Authors:** Hanoch Goldshmidt, Devorah Matas, Anat Kabi, Shai Carmi, Ronen Hope, Shulamit Michaeli

**Affiliations:** The Mina & Everard Goodman Faculty of Life Sciences, and Advanced Materials and Nanotechnology Institute, Bar-Ilan University, Ramat-Gan, Israel; University of Wisconsin-Madison, United States of America

## Abstract

Trypanosomes are parasites that cycle between the insect host (procyclic form) and mammalian host (bloodstream form). These parasites lack conventional transcription regulation, including factors that induce the unfolded protein response (UPR). However, they possess a stress response mechanism, the spliced leader RNA silencing (SLS) pathway. SLS elicits shut-off of spliced leader RNA (SL RNA) transcription by perturbing the binding of the transcription factor tSNAP42 to its cognate promoter, thus eliminating *trans*-splicing of all mRNAs. Induction of endoplasmic reticulum (ER) stress in procyclic trypanosomes elicits changes in the transcriptome similar to those induced by conventional UPR found in other eukaryotes. The mechanism of up-regulation under ER stress is dependent on differential stabilization of mRNAs. The transcriptome changes are accompanied by ER dilation and elevation in the ER chaperone, BiP. Prolonged ER stress induces SLS pathway. RNAi silencing of SEC63, a factor that participates in protein translocation across the ER membrane, or SEC61, the translocation channel, also induces SLS. Silencing of these genes or prolonged ER stress led to programmed cell death (PCD), evident by exposure of phosphatidyl serine, DNA laddering, increase in reactive oxygen species (ROS) production, increase in cytoplasmic Ca^2+^, and decrease in mitochondrial membrane potential, as well as typical morphological changes observed by transmission electron microscopy (TEM). ER stress response is also induced in the bloodstream form and if the stress persists it leads to SLS. We propose that prolonged ER stress induces SLS, which serves as a unique death pathway, replacing the conventional caspase-mediated PCD observed in higher eukaryotes.

## Author Summary

Trypanosomes are the causative agent of major parasitic diseases such as African sleeping sickness, leishmaniasis and Chagas' disease that affect millions of people mostly in developing countries. These organisms diverged very early from the eukaryotic linage and possess unique molecular mechanisms such as *trans*-splicing and RNA editing. Trypanosomes lack polymerase II promoters that govern the transcription of protein coding genes. Eukaryotes respond to unfolding of proteins in the endoplasmic reticulum (ER) by a distinct transcriptional programming known as the unfolded protein response (UPR). In this study, we demonstrate that despite the lack of transcriptional regulation, procyclic trypanosomes change their transcriptome as a response to ER stress by differential mRNA stabilization. Prolonged ER stress induces a unique process, the spliced leader RNA silencing (SLS), that shuts off the *trans*-splicing and the production of all mRNAs. SLS is induced both by prolonged ER stress and by knock-down of factors involved in ER translocation in both life stages of the parasite. SLS induces programmed cell death (PCD) evident by the hallmark of apoptosis in metazoa (DNA fragmentation, membrane flipping and ultrastructural changes). We propose that SLS serves as a unique death pathway replacing the conventional caspase-mediated PCD observed in higher eukaryotes.

## Introduction

The endoplasmic reticulum (ER) is the site for folding of proteins that traverse the ER on the way to the outer membrane or internal organelles of the vesicular transport machinery. The ER is also the site of lipid synthesis and it maintains the cellular calcium homeostasis [1]. The ER performs these functions using resident chaperones, high levels of calcium and an oxidating environment. Proteins that translocate to the ER undergo proper folding and those can exit the ER. However, if the protein fails to fold properly, it is exported back to the cytoplasm and is degraded by the proteasome [2],[3]. ER function is sensitive to changes in calcium level, inhibition of glycosylation, oxidative stress, and exposure to reducing agents. These conditions induce the ER stress response, which triggers specific signaling pathways including the unfolded proteins response (UPR), which leads to reduction in the load of proteins to be translocated, enhanced degradation of misfolded proteins, and increased folding capacity of the ER [3]–.

In yeast, UPR is dependent on IRE1, which is a bi-functional kinase and endonuclease that cleaves a non-conventional intron from HAC1 mRNA, which encodes a transcription factor (bZIP) [6],[7]. Homologues proteins exist in mammals; IRE1 cleaves an intron in XBP1 encoding a potent bZIP transcription factor. This transcription factor activates genes essential for executing the UPR [8]. In mammals, two additional ER transmembrane proteins, PERK and ATF6, participate in UPR. PERK phosphorylates eIF2α to mediate translational attenuation. ATF6 is cleaved in the Golgi and translocates to the nucleus to activate the transcription of chaperones, such as BiP [9]–[12]. When the ER stress is persistent, the cells activate the apoptotic pathway via the activation of Caspase 12 [13],[14].

Trypanosomatids are known for their non-conventional gene expression mechanisms, including *trans*-splicing [15],[16] and RNA editing [17],[18]. *Trans*-splicing is required for the maturation of all mRNAs in these parasites. In this process, a small exon, the spliced leader (SL), encoded by a small RNA, the SL RNA, is donated to all pre-mRNA by *trans*-splicing [16]. No promoters upstream to protein coding genes transcribed by polymerase II were identified. Protein coding genes are arrayed in long polycistronic transcription units which are processed post-transcriptionally by the concerted action of *trans*-splicing and polyadenylation [15]. It is believed that gene expression in these parasites is regulated primarily post-transcriptionally at the level of mRNA degradation and translation; for most genes, the signals that dictate this regulation are situated at the 3′ UTR [19]. However, trypanosomes do not completely lack transcriptional regulation. The SL RNA has a defined promoter that binds specific transcription factors such as tSNAPc [20]–[22].

Our collective knowledge of the regulation of trypanosome gene expression is consistent with the novel stress-induced regulatory mechanism that controls the transcriptome under variety of stresses; this mechanism was named SLS, for SL RNA silencing [23]. SLS was discovered in cells silenced for the signal recognition particle receptor, SRα. Depletion of SRα results in a dramatic decrease in the level of SL RNA, leading to major reduction in mRNAs. Nascent transcription in permeable cells showed that SL RNA transcription is specifically extinguished under these conditions. ChIP assay further demonstrated that under SRα depletion, the SL RNA transcription factor, tSNAP42, fails to bind to the SL RNA promoter. The hallmark of SLS is therefore shut-off of SL RNA transcription, followed by massive accumulation of tSNAP42 in the nucleus [23].

Most recently, a very detailed study on the trypanosome transcriptome was performed and revealed significant changes between procyclic and bloodstream form trypanosomes. However, the same study failed to detect changes generally elicited by classical UPR inducers or changes that are generally elicited under serum starvation [24]. It is currently unknown whether trypanosomes lack the UPR mechanism, which is replaced by SLS in these organisms, or whether both mechanisms exist, and SLS is activated to induce PCD when UPR fails. Accumulated data suggest that PCD exists in trypanosomatids, and plays a major role in maintaining and regulating the parasite population [25],[26]. In metazoa, type I PCD is induced by caspases. Effector caspases activate proteases and nucleases, eventually leading to apoptosis. Trypanosomes lack homologues to metazoan caspases, but have five metacaspases [27]–[29]. However, up till now, it is believed that these metacaspases are not involved in apoptosis [27],[29]. In recent years, PCD pathways that are caspase-independent, such as autophagy were shown to exist. Autophagy functions in protein degradation and organelle turnover during stresses, such as nutrient and growth factor deprivation, to assure cell survival. Autophagy machinery can be also recruited to kill cells under certain conditions, generating a caspase-independent form of PCD [30]. Recent studies indicate that trypanosomatids possess such an autophagic pathway [31],[32].

In this study, we demonstrate that despite the lack of IRE1/XBP1 homologues that mediate the UPR in all eukaryotes, trypanosomes change their transcriptome in response to ER stress much like yeast and metazoa. The mechanism of ER stress response is unique, and is based on stress-induced mRNA stabilization of mRNAs, which are essential for executing the response. SLS is triggered under prolonged ER stress induced either by chemicals or by silencing of SEC63 and SEC61- two major components of ER translocation machinery. Typical hallmarks of PCD such as DNA fragmentation, exposure of phosphatidyl serine, increase in cytosolic calcium, reduction in mitochondria membrane potential and ROS production, were observed in SLS-induced cells. We propose that SLS is the apoptotic branch induced under persistent ER stress that leads to programmed cell death in both procyclic and bloodstream form trypanosomes.

## Results

### The ER stress response in trypanosomes: Transcriptome changes in *T. brucei* treated with Dithiothreitol (DTT) resemble UPR in other eukaryotes

**Figure 1:**
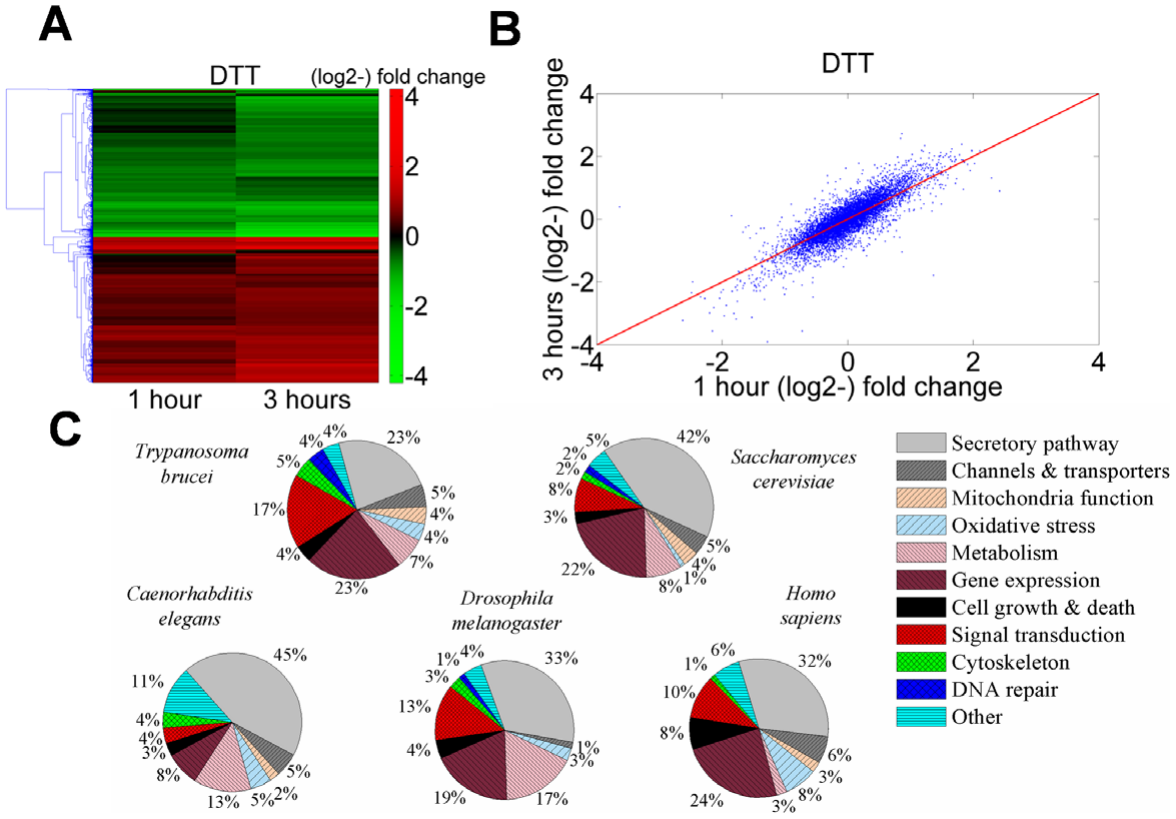
Transcriptome changes in *T. brucei* treated with DTT resemble analogous changes in other eukaryotes. **A.** Heat map of genes that are differentially regulated in cells treated with 4 mM DTT for 1 or 3 hrs relative to untreated cells. Transcripts that differ from the control by >1.5-fold change were chosen for the analysis. Each column represents the average of three biological replicates. The heat map was generated using the average linkage hierarchical clustering algorithm and Euclidean distance as a similarity measure. The diagram represents the differential expression level according to the following color scale: red- up-regulated genes; green- down-regulated genes. **B.** Scatter plot analysis showing correlation of the transcriptome data from trypanosomes treated with 4 mM DTT for 1 or 3 hours. **C.** Pie diagram of up-regulated genes during UPR in four organisms (Table S3), compared to the response in *T. brucei* (Table S2). Data were obtained for the following organisms. *S. cerevisiae* [3]; *C. elegans*: [33]; *D. melanogaster* [34]; *H. sapiens:* [35] (see Table S3).

To examine whether procyclic stage trypanosomes possess an ER stress response, the transcriptome was examined for up and down-regulation upon Dithiothreitol (DTT) treatment. RNA was prepared from cells treated with 4 mM DTT for 1 and 3 hours and the transcriptome was compared to untreated cells. The Lowess-normalized data were used to identify genes whose expression appeared to be significantly (*P*<0.05) up-regulated with an arbitrary cutoff of 1.5 fold. A list of these genes is provided in Table S2. In order to visualize the difference in gene expression between normal and under ER stress, we compared the changes of transcriptome after 1 and 3 hours of treatment. Hierarchical clustering was performed on the regulated genes, and a heat map was constructed (Fig. 1-A). The results demonstrate that the changes in the transcriptome between 1 and 3 hrs of treatment were similar in both up- and down-regulated genes (r = 0.86), indicating that the changes were treatment-specific (Fig. 1-B). Moreover, the amplitude of the up and down-regulation was increased by 1.24 fold when comparing the differential expression from 1 to 3 hours of treatment.

Inspection of the up-regulated genes (Table S2) suggests that many genes which are involved in the core processes of classic UPR, namely, protein folding, degradation, translocation, sorting, and lipid metabolism, were up-regulated. Additionally, genes involved in mitochondrial functions, redox balance, metabolism, cytoskeleton, and movement were also up-regulated. Interestingly, a large number of genes involved in signal transduction and gene expression were affected, as well.

To compare the response of trypanosomes under ER stress to that of other eukaryotes, microarray data from UPR induced cells of *Saccharomyces cerevisiae* [3], *Caenorhabditis elegans* [33], *Drosophila melanogaster* [34], and *Homo sapiens* [35] were downloaded and analyzed. Genes found to be up-regulated were classified into the same functional categories (Table S3) that were already defined for the trypanosome transcriptome (Table S2). Note that for *S. cerevisiae*, *C. elegans*, and *D. melanogaster*, the list of up-regulated genes was refined by filtering out genes whose expression under ER stress was not XBP-1 or IRE-1 dependent and therefore are not directly related to the UPR response. In trypanosomes and humans, no such filtering was possible.

The results presented as pie diagrams in Figure 1-C, demonstrate that in trypanosomes, as in all other organisms, the largest category of up-regulated genes in response to ER stress is the category of genes that function in protein secretion. Moreover, except movement, all the other functions that were shown to be up-regulated in trypanosomes are also up-regulated in some if not all other organisms. Changes in motility are specific to trypanosomes. Of note is also that a significantly high fraction of the down-regulated genes (35%, *P*<10^−6^) encode for proteins destined to traverse the ER (i.e., harboring either a signal-peptide or trans-membrane domains), similarly to *D. melanogaster* [34].

### The response to ER stress is mediated by stress-induced mRNA stabilization

**Figure 2:**
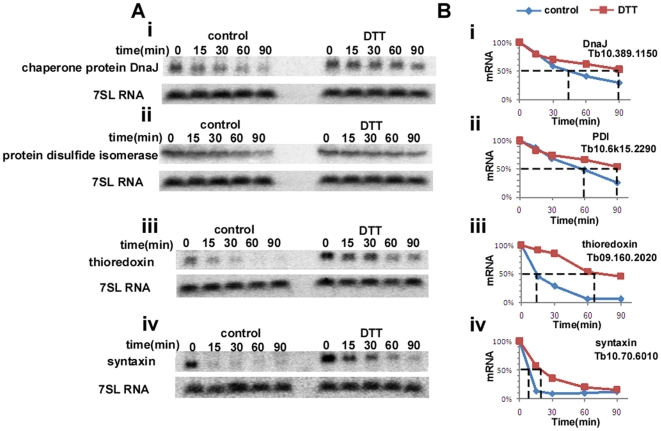
ER-stress response regulates the half-life of mRNAs. **A.** Northern analysis of mRNA levels. Untreated cells or cells treated with 4 mM DTT for 1.5 hours were treated with sinefungin (2 µg/ml) and, after 10 min, with actinomycin D (30 µg/ml). RNA was prepared at the time points indicated above the lanes, separated on a 1.2% agarose–formaldehyde gel, and subjected to Northern analysis with the indicated gene-specific probes. 7SL RNA was used to control for equal loading. **i**, chaperone protein DNAJ; **ii**, protein disulfide isomerase; **iii**, thioredoxin; **iv**, syntaxin. **B.** Half life of the different mRNAs in untreated versus DTT-treated cells. The hybridization signals were measured by densitometry. The level of the mRNAs of **i**, chaperone protein DNAJ; **ii**, protein disulfide isomerase; **iii**, thioredoxin; **iv**, syntaxin; in the different time points is illustrated by blue diamonds (untreated) and by red squares (DTT treatment). The half-life is indicated by the broken lines.

Trypanosomes lack conventional transcription regulation, and therefore it is expected that their genome lacks homologues of IRE1/XBP1. Since mRNA stability is a very dominant mechanism of regulation, we examined the possibility that up-regulation results from mRNA stabilization during ER stress. To this end, procyclic stage trypanosomes (untreated) and treated with DTT for 90 minutes, were treated with sinefungin and actinomycin D, inhibitors of *trans*-splicing and transcription, respectively [36]. Incubation was for increasing time points to measure possible changes in the half life of mRNAs during ER stress. RNA was extracted and subjected to Northern analysis with probes specific to genes whose level was significantly changed upon DTT treatment and that were shown to also be induced in other organisms. The results in Figure 2 demonstrate that the half-life of DNAJ, protein disulfide isomerase (PDI), thioredoxin and syntaxin were all increased (from 45 to 90 min, 60 to 90 min, 13 to 65 min and 10 to 20, respectively) under DTT treatment, suggesting that the up-regulation of these transcripts during ER stress can be explained by stress-induced mRNA stabilization. The level of mRNAs that were unchanged during DTT treatment (based on microarray analysis) was also examined, and no changes in their stability was observed upon the ER stress (results not shown), indicating the specificity of this regulation.

### ER stressors elicit ER dilation and over expression of the ER chaperone, BiP

**Figure 3:**
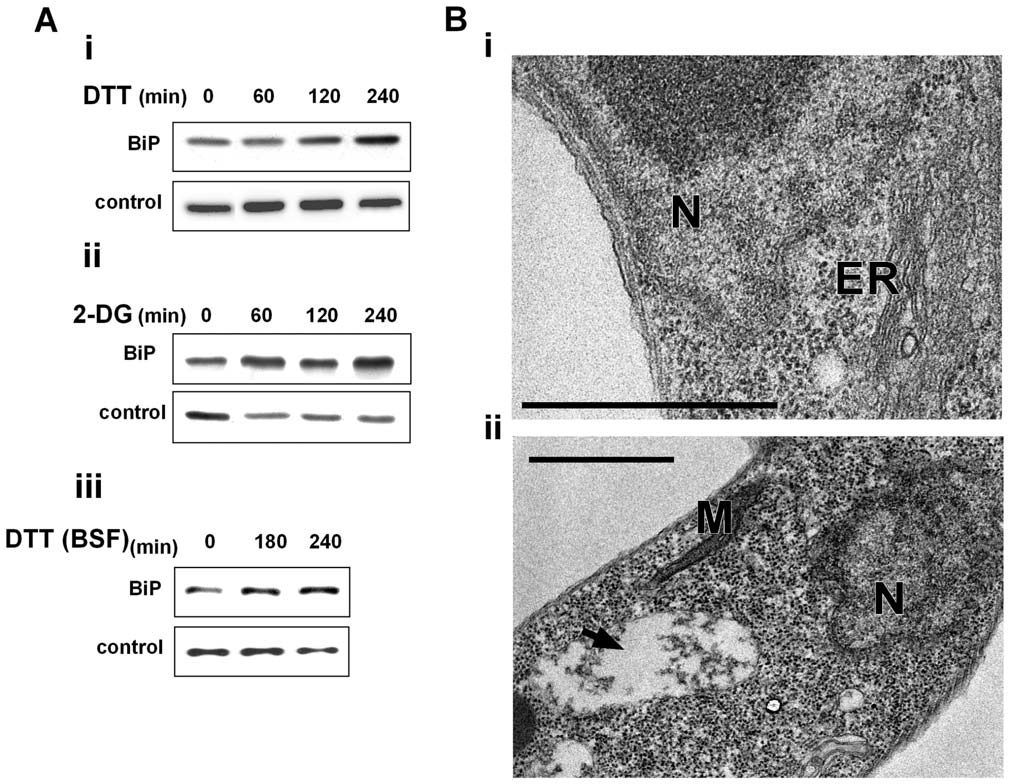
ER stressors induce BiP expression and ER dilation. A. Western blot analysis of BiP protein. Whole cell extracts (10^6^ cells/lane) were prepared from procyclic (**i**-**ii**) or bloodstream trypanosomes (**iii**) treated with ER stressors for different time points. The extracts were separated on a 10% SDS-polyacrylamide gel and subjected to Western blot analysis using anti-BiP antibodies. hnRNPD_0_ was used to control loading. **i.** 4 mM DTT; **ii.** 20 mM 2-deoxy-D-glucose (2-DG); **iii.** 4 mM DTT. **B.** Electron micrographs. **i.** untreated cells; **ii.** cells treated with 4 mM DTT for 1 hour. The black arrow indicates the expanded ER. N, nucleus; M, mitochondria; ER, endoplasmic reticulum. Scale bars, 1 µm.

One of the hallmarks of UPR in yeast and metazoa is the ability to induce the production of ER chaperones to increase the folding capacity of the ER [1],[3],[4],[37]. Induction of chaperones is not only mediated by increase in the transcription but also by preferential translation and proteolysis under stress [38]. Although BiP mRNA is not among the mRNAs whose level is most highly increased under ER stress ([3],[39] and this study) its elevation at the protein level serves as a hallmark for UPR. To examine if in trypanosomes BiP is increased upon induction of ER stress, its level was examined after treating cells with DTT or 2-deoxy D- glucose (2-DG), which affects glycosylation [40],[41]. Although 2-DG inhibits glycosylation and is known to induce the UPR response in other eukaryotes, it may also have other effects on carbon source sensing and metabolism [41]. The results presented in Figure 3-Ai and Figure 3-Aii suggest that both treatments result in increased levels of BiP in a time-dependent manner. Next, we examined if ER stress response can be induced also in the bloodstream form. The results (Fig. 3-Aiii) suggest that under the conditions used, BiP level was increased. These results are contrast to those previously published [24]. However, note that we used 4mM DTT, whereas the previous study used only 1 mM DTT. The results suggest that ER stress response operates in both stages of the parasite.

To observe cellular changes that may accompany response to ER stress, ultrastructure analysis using electron microscopy was performed. After 1 hour of DTT treatment, the most prominent change observed by transmission electron microscopy (TEM) is the expansion of the ER (compare Fig. 3 Bi to Bii). ER expansion was previously observed in yeast and mammals, and it was suggested that the increase in the ER may help to accommodate newly synthesized ER proteins and inhibit aggregation of unfolded proteins by reducing their concentration [42],[43]. The ER lumen was not only dilated, but seemed to contain dense material that might correspond to aggregated proteins (Fig. 3-Bii).

### ER stress induces irreversible growth arrest and SLS

**Figure 4:**
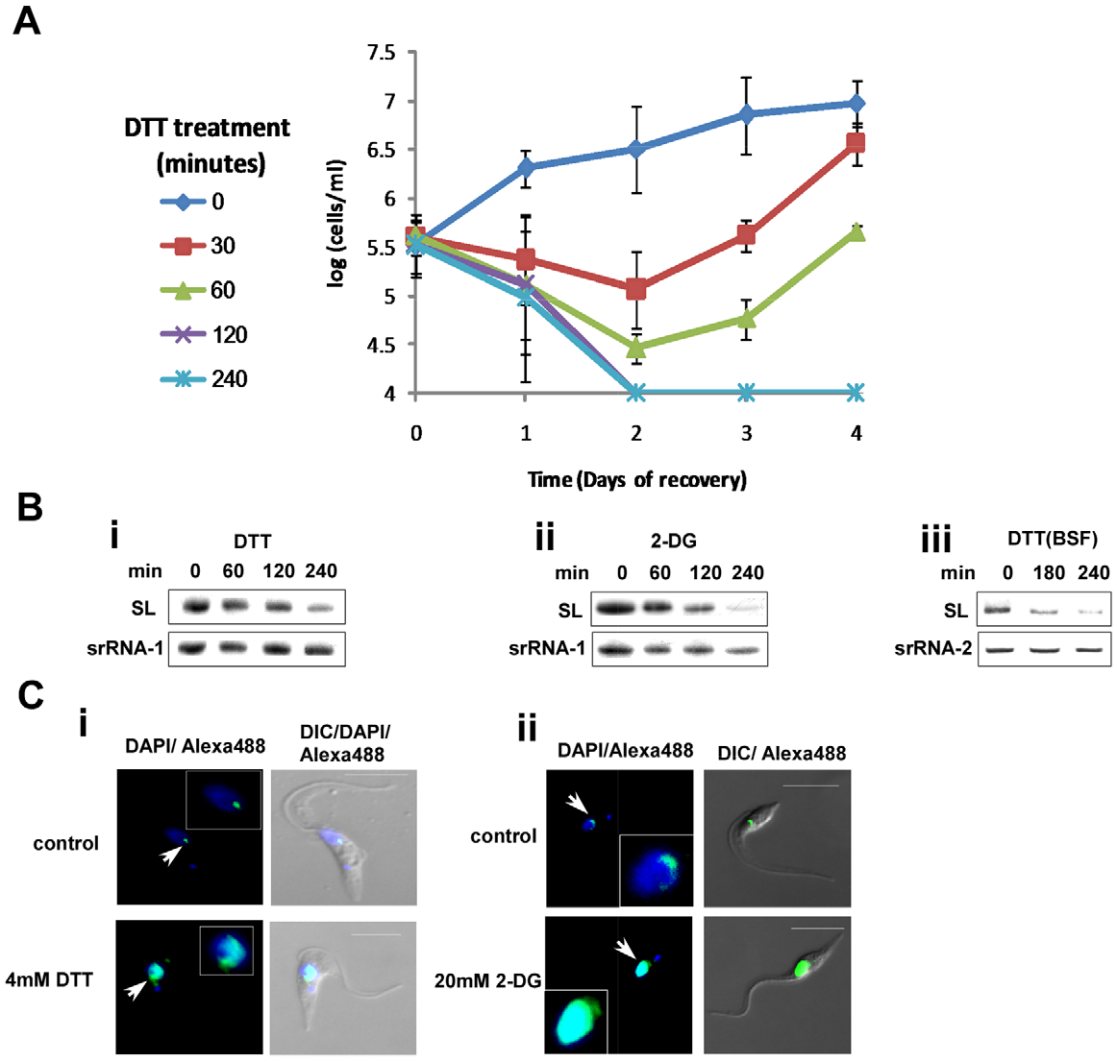
Cell death and induction of SLS by ER stressors. **A.** Recovery of cells after DTT treatment. Cells were treated with 4 mM DTT for 0, 30, 60, 120 and 240 minutes, washed twice in PBS and suspended in growth media to the same concentration. Cell growth was monitored for 4 days. Symbols indicate the duration of DTT treatment. **B.** Northern blot analysis of RNA from cells treated with ER stressors. RNA (10 µg) was prepared from procyclic (1–2) or bloodstream trypanosomes (3) treated with **i.** 4 mM DTT; **ii.** 20 mM 2-deoxy-D-glucose for 0, 60, 120 or 240 minutes or **iii.** 4 mM DTT for 0, 180, 240 minutes and was subjected to Northern analysis with radio-labeled anti-sense RNA probe. The srRNA-1 or srRNA-2 probes were used for loading control. **C.** Immunofluorescence monitoring the changes in amount and localization of tSNAP42 upon treatment with ER stressors. Cells were treated with **i.** 4 mM DTT; **ii.** 20 mM 2-DG for 3 hours. The cells were fixed with 4% (v/v) formaldehyde for 25 min, incubated with tSNAP42 antibodies and detected as described in Materials and Methods. The nucleus was stained with DAPI. DIC- differential interference contrast. The localization of tSNAP42 is indicated with arrows. Scale bars. 5 µm. Enlargement of the nuclear area is framed.

To examine if trypanosomes can recover following removal of ER stress, procyclic stage parasites were exposed to DTT for different time periods ranging from 30 to 240 minutes. After incubation with DTT, the DTT was washed out, and growth was monitored. The results (Figure 4-A) suggest that after exposure to DTT for 120 minutes or more, cells were unable to resume growth upon withdrawal of DTT, suggesting that prolonged exposure to DTT leads to cell death.

SLS was shown to be induced in procyclic stage trypanosomes upon silencing of SRP receptor or low pH [23]. We previously proposed that SLS might replace the conventional ER stress response because of the lack of conventional transcriptional regulation that would be required to induce the production of mRNAs needed for this process. The results in Figures 1–3 suggest that trypanosomes possess an ER stress response, and it was therefore of great interest to examine if this response is associated with SLS. SLS is characterized by two hallmarks, reduction in SL RNA level and changes in the amount and localization of tSNAP42. The results in Figure 4-Bi and Figure 4-Bii suggest that SLS is induced 180 and 120 minutes after exposure to DTT or 2-DG, respectively. SLS is induced after BiP induction, since induction of BiP was detected 120 minutes after DTT addition (Fig. 3-Ai) and 60 minutes after 2-DG addition (Fig. 3-Aii), suggesting that the ER stress response is induced before SLS. To examine if prolonged ER stress induces SLS also in bloodstream trypanosomes, SL RNA level was examined upon treatment with DTT. The results in (Fig. 4-Biii) demonstrate the induction of SLS in bloodstream trypanosomes after 180 minutes exposure to DTT.

The induction of SLS is clearly observed when comparing the localization of the SL RNA transcription factor, tSNAP42. Under normal conditions, tSNAP42 is confined to the nuclear domain where SL RNA is transcribed and assembled with the Sm core proteins [44],[45]. Under prolonged ER stress, the level of tSNAP42 increases, filling up the entire nucleus (Fig. 4-C). These data suggest that SLS is activated after the response to ER stress was initiated; 180 minutes (in DTT-treated cells) or 120 minutes (in 2-DG-treated cells).

### Silencing of functions involved in ER translocation induce SLS in both stages of the parasite

**Figure 5:**
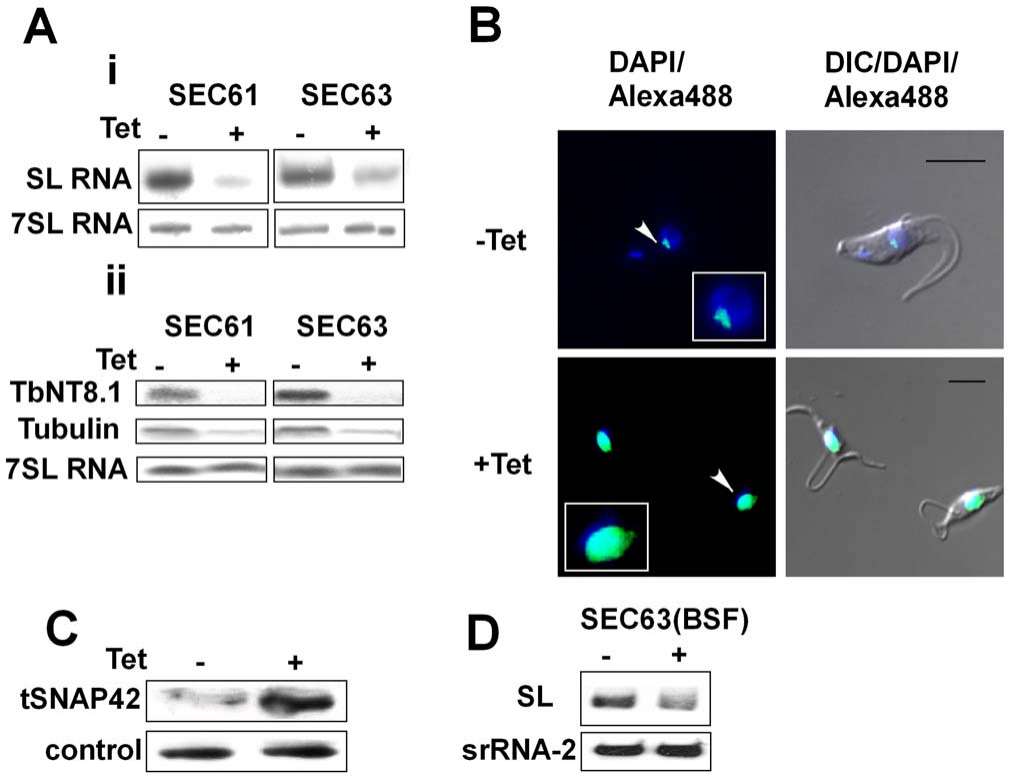
Silencing of SEC61 and SEC63 induces SLS. **A.** Northern blot analysis of SEC63 and SEC61 RNAs. RNA was prepared from uninduced (-Tet) and silenced cells 3 days after induction (+Tet) and subjected to Northern analysis with radio-labeled probes. **i.** Small RNAs; **ii.** mRNAs. The transcripts examined are indicated. **B.** Immunofluorescence monitoring the changes in amount and localization of tSNAP42 during SEC63 silencing. Uninduced cells (-Tet) and cells induced (+Tet) for 48 hours were fixed with 4% (v/v) formaldehyde for 25 min, incubated with tSNAP42 antibodies and detected as described in Materials and Methods. The nucleus was stained with DAPI. DIC- differential interference contrast. The localization of tSNAP42 is indicated with arrows. Scale bars, 5 µm. Framed region shows enlargement of the nuclear area. **C.** Changes in the level of tSNAP42 in cells carrying SEC63 silencing construct. Western blot analysis using anti-tSNAP42 antibodies. Nuclei were extracted from uninduced cells (-Tet) and cells induced (+Tet) for 48 hours, as previously described [23]. The extracts were separated on 10% SDS-PAGE and reacted with anti tSNAP42 antibodies. Rabbit anti PTB1 antiserum was used as a loading control. **D.** Northern blot analysis of RNA from bloodstream trypanosomes silenced for SEC63. RNA was prepared from uninduced (-Tet) and silenced cells 3 days after induction (+Tet) and subjected to Northern analysis with radio-labeled probes. srRNA-2 probe was used for loading control.

We previously demonstrated that SLS is induced following silencing of the SRP receptor in the procyclic stage [23]; it was therefore of interest to examine whether SLS is induced following other perturbations that are connected to ER translocation functions. For this analysis, we chose to investigate and compare three functions that control translocation across the ER membrane, SRα, SEC63 and SEC61. We have previously demonstrated that SEC63 is essential for translocation of proteins to the ER via both pathways - the co-translational pathway mediated by SRP and the post-translational pathway mediated by chaperones [46]. The SEC61 protein constitutes the translocon. SEC61 was silenced using a stem-loop construct as described in the Materials and Methods. The results in Figure 5-Ai demonstrate that upon silencing of SEC61 or SEC63, the level of SL RNA was reduced, with no change in the level of 7SL RNA. The reduction elicited a major decrease in the level of mRNAs due to inhibition of *trans*-splicing (Fig. 5-Aii). The reduction in SL RNA was accompanied by the classical nuclear accumulation of tSNAP42 (Fig. 5-B). Indeed the level of tSNAP42 was increased upon SEC63 silencing as observed in Figure 5-C. The results suggest the perturbation in ER translocation due to elimination of SEC61 or SEC63 elicits SLS, much like that observed in SRα silenced cells [23]. To examine if SEC63 silencing also induces SLS in bloodstream form, transgenic parasites expressing a T7 opposing construct to silence the SEC63 gene (Figure S2) were generated as described in Materials and Methods. The results (Figure 5-D) demonstrate that silencing of SEC63 induces SLS as evident by marked reduction in the level of SL RNA.

### DNA is fragmented in SLS-induced cells

**Figure 6:**
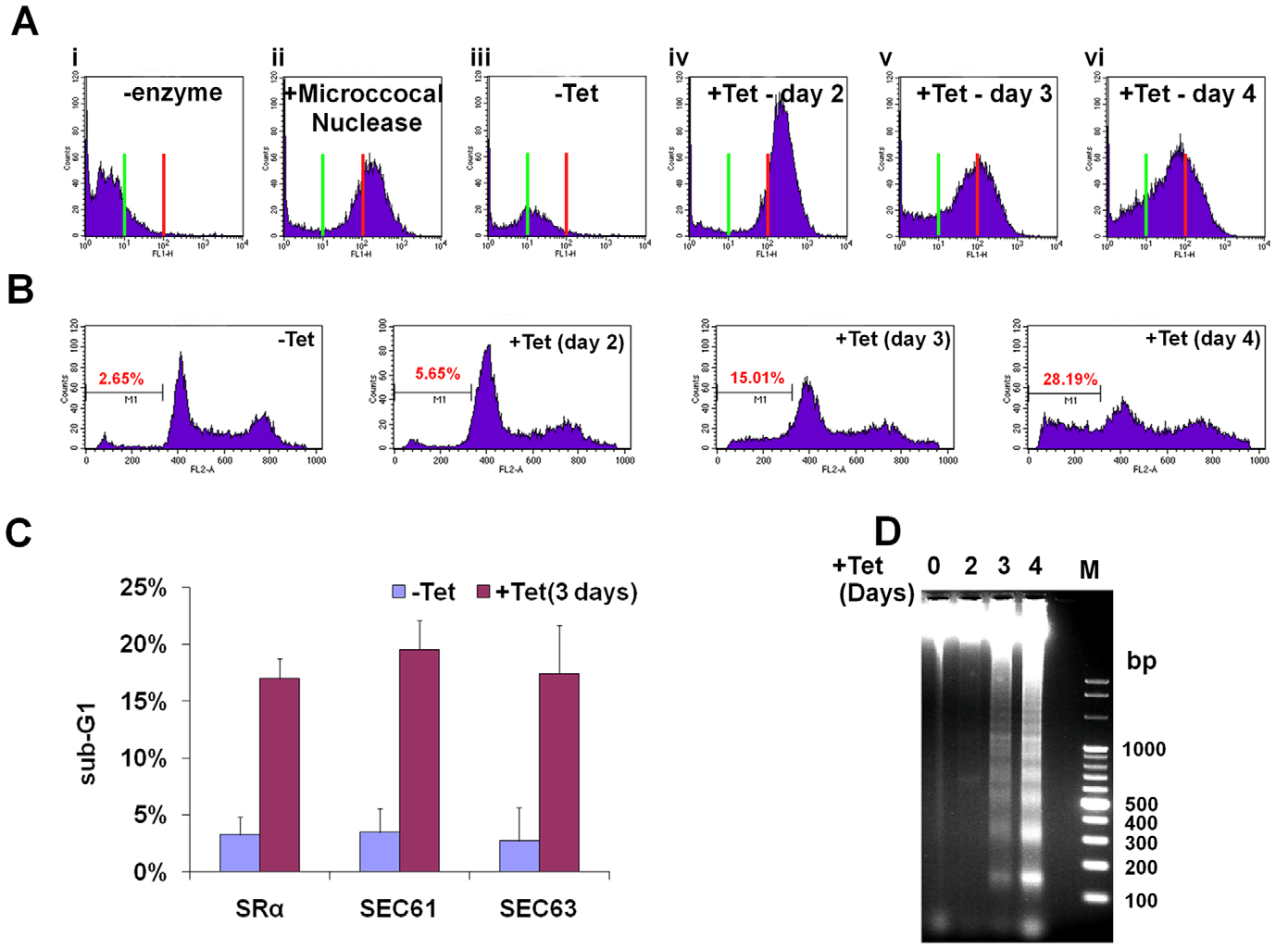
SLS elicits DNA fragmentation. **A.** TUNEL assay in SRα silenced cells. TUNEL assay and FACS analysis were performed as described in Materials and Methods. Cells after 3 days of induction without addition of terminal deoxynucleotidyl transferase was used as a negative control. **ii.** Uninduced cells treated for 10 min with 3000 units/ml of micrococcal nuclease before labeling, used as a positive control. **iii.** Uninduced cells (-Tet). **iv-vi.** SRα silenced cells for 2, 3 or 4 days (+Tet), respectively. The green line indicates fluorescence level of cells harboring undamaged DNA. Red line indicates the fluorescence level of cells carrying damaged DNA. **B.** Analysis of DNA fragmentation by propidium iodide staining and flow cytometry. Uninduced cells (-Tet) or SRα cells silenced for 2, 3 or 4 days (+Tet) were fixed with 100% ethanol for 12 hours at 4°C, washed with PBS and stained for 0.5 hour with 25 µg/ml propidium iodide in the presence of 1 µg/ml RNaseA. DNA content was measured by FACS. The percentage of sub-G1 cells (M1) is indicated. **C.** Analysis of DNA fragmentation by propidium iodide staining and flow cytometry. The graph represents measurements of sub-G1 population from SRα, SEC61 and SEC63 cells, uninduced (-Tet) or silenced for 3 days(+Tet). The data are derived from three independent experiments and the S.D is indicated. **D.** DNA laddering during SRα silencing. DNA was isolated from uninduced cells or cells silenced for 2, 3 or 4 days (+Tet) as described in Materials and Methods. DNA (10 µg) was separated on a 1% agarose gel and the gel was stained with ethidium bromide. M - 100bp DNA ladder.

Is SLS a programmed cell death pathway that is induced under prolonged ER stress much like apoptosis in mammalian cells? To examine this intriguing possibility, the hallmarks of programmed cell death were investigated in SLS-induced procyclic stage trypanosomes. First, the DNA fragmentation during SLS was measured by TUNEL. TUNEL identifies nicks in DNA by measuring the incorporation of fluorescently labeled dUTP using terminal deoxynucleotidyl transferase (TdT). The results presented in Figure 6-A demonstrate an increase in free ends of DNA, marking DNA fragmentation in SRα silenced cells. Two controls were used to demonstrate the specificity of the assay; in the absence of TdT no signal was observed (Fig. 6Ai), and maximal signal was obtained upon treatment with micrococcal nuclease, which induces DNA breaks (Fig. 6Aii). Another assay to monitor DNA fragmentation is by propidium iodide (PI) staining and flow cytometry. Apoptotic cells display a broad hypodiploid (sub-G1) peak, which can be easily discriminated from the narrow peak of cells with normal (diploid) DNA content. SRα silenced cells were examined for the content of sub-G1 DNA upon silencing. The results (Fig. 6-B) demonstrate an increase in the number of sub-G1 cells, suggesting that many cells in the population undergo DNA fragmentation and lose DNA fragments as response to SRα silencing. The same experiment was performed for all silencing treatments that were shown to induce SLS, and the results in Figure 6-C indicate increase in sub-G1 population in cells silenced for SRα, SEC61 and SEC63. The TUNEL and sub-G1 assays can also detect DNA fragmentation in necrotic cells. The inter-nucleosomal DNA digestion by endogenous nucleases to yield a characteristic laddering pattern is one of the hallmarks of apoptotic cells that does not take place in necrotic cells [47]. Laddering of DNA is one of the final steps in the apoptotic process, together with chromatin condensation, nuclear picnosis and the formation of apoptotic bodies. The results in Figure 6-D demonstrate DNA laddering in SRα silenced cells, and that laddering is increased during SLS induction. The maximal laddering is observed 4 days after the induction of silencing. Identical DNA laddering was observed under SEC63 silencing (data not shown). In sum, the results demonstrate that SLS induction leads to DNA fragmentation and laddering, suggesting that SLS-induced procyclic stage trypanosomes undergo PCD.

### Phosphatidyl serine (PS) exposure in SLS induced procyclic stage trypanosomes

**Figure 7:**
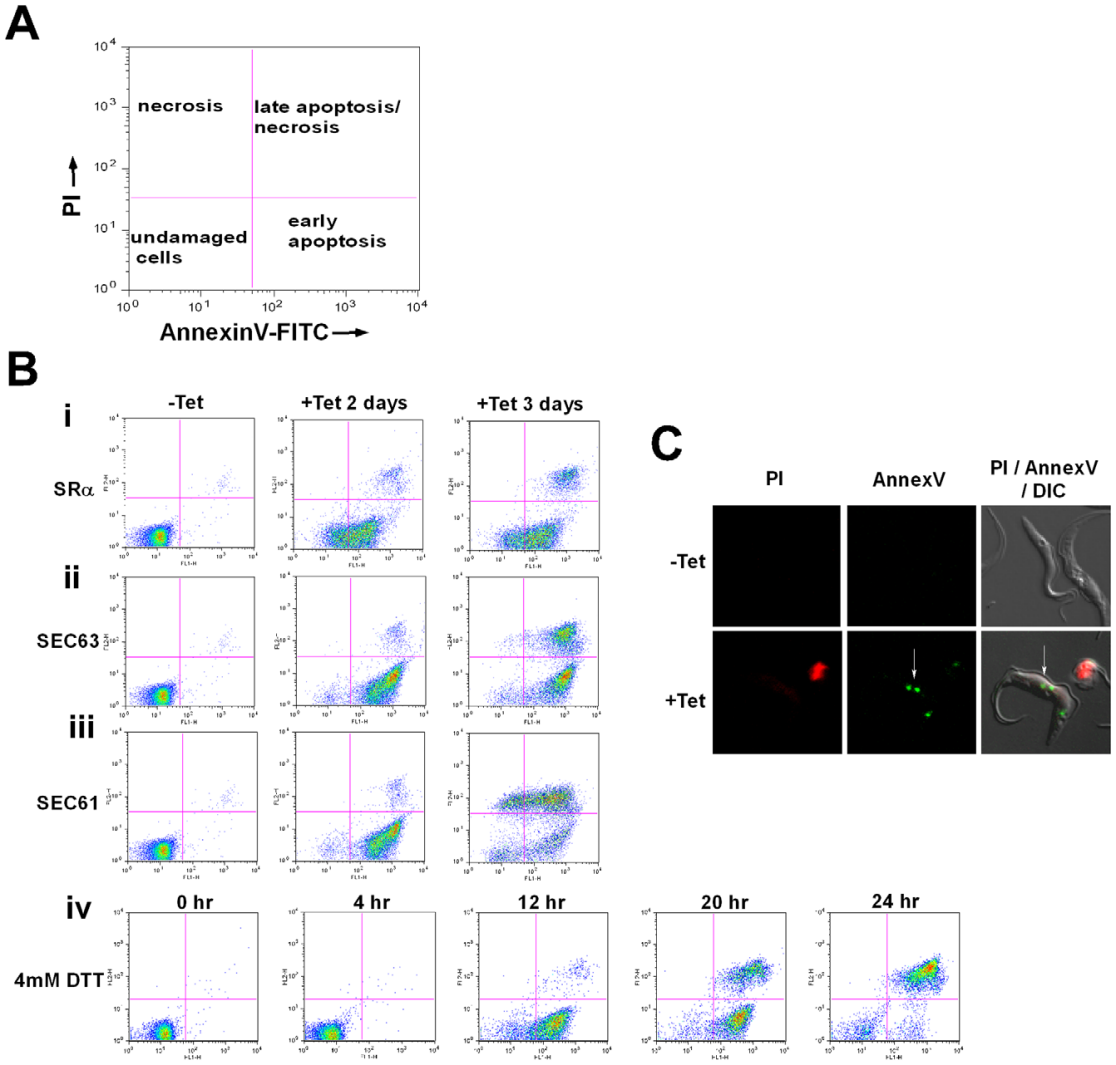
Phosphatidylserine exposure during SLS induction. **A.** Diagram showing the different cell populations detected by staining with AnnexinV and PI. **B.** Analysis of exposed phosphatidylserine on outer membrane of SLS induced cells. Uninduced cells (-Tet) or cells silenced for 2 and 3 days (+Tet), and cells treated with DTT for 0, 4 12, 20 and 24 hours were reacted with fluorescein isothiocyanate-labeled AnnexinV antibodies (MBL^©^) and stained with propidium iodide according to the manufacturer's instructions. The cells were analyzed by FACS. The panels represent **i.** SRα silenced cells; **ii.** SEC61 silenced cells; **iii.** SEC63 silenced cells; **iv.** DTT treated cells. **C.** AnnexinV - PI staining of SLS induced cells. SRα cells were silenced for 3 days (+Tet). Uninduced and silenced cells were stained with AnnexinV and PI, fixed with 4% formaldehyde and visualized. The exposed phosphatidylserine in propidium iodide negative cells are marked by a white arrow.

Induction of apoptosis causes externalization of phosphatidyl serine (PS) on the surface of the apoptotic cells. AnnexinV binds to the exposed PS of apoptotic cells. The dual staining with propidium iodide (PI) enables the distinction between necrotic and apoptotic cells [48]. As indicated in Figure 7-A, necrotic cells lose their membrane integrity and become permeable to PI (Figure 7-A, upper left panel). Live cells are stained neither with AnnexinV nor with PI (bottom left panel). During early apoptosis, PS is exposed on the surface but because of the integrity of the plasma membrane the cells are not be stained with PI (bottom right panel). Late apoptotic or necrotic cells lose their membrane integrity and are stained with both PI and AnnexinV (upper right panel). SLS induced cells - elicited by silencing of SRα, SEC63 and SEC61 or after treatment with DTT - were stained with AnnexinV before the cells became permeable to PI, indicating that PS was externalized. These data suggest that apoptosis is induced 2 days after induction of silencing of each of the factors (Fig. 7-Bi-iii, middle column), or 12 hrs after DTT treatment (Fig. 7-Biv). After 3 days of silencing, the apoptotic cells also became permeable to PI (Fig. 7-Bi-iii, right column). Cells treated with DTT for 20 or 24 hrs also became permeable to PI (Fig. 7-Biv). To visualize cells externally stained by AnnexinV, cells were incubated with AnnexinV and PI, and visualized by fluorescence microscopy. The results in Figure 7-C demonstrate that although the cells were not stained by PI, AnnexinV staining was observed only in the silenced cells and not in control, uninduced cells. The results suggest that at early time points of silencing, PS became externally exposed on the cells, most probably as a result of membrane flipping, a signature of apoptosis. Note that, PS exposure was not observed in cells silenced either for SmD1 [49] or PTB2 [50] that are dying as a result of silencing (Figure S1-A).

### Cytoplasmic Ca^2+^ increases in SLS induced cells

**Figure 8:**
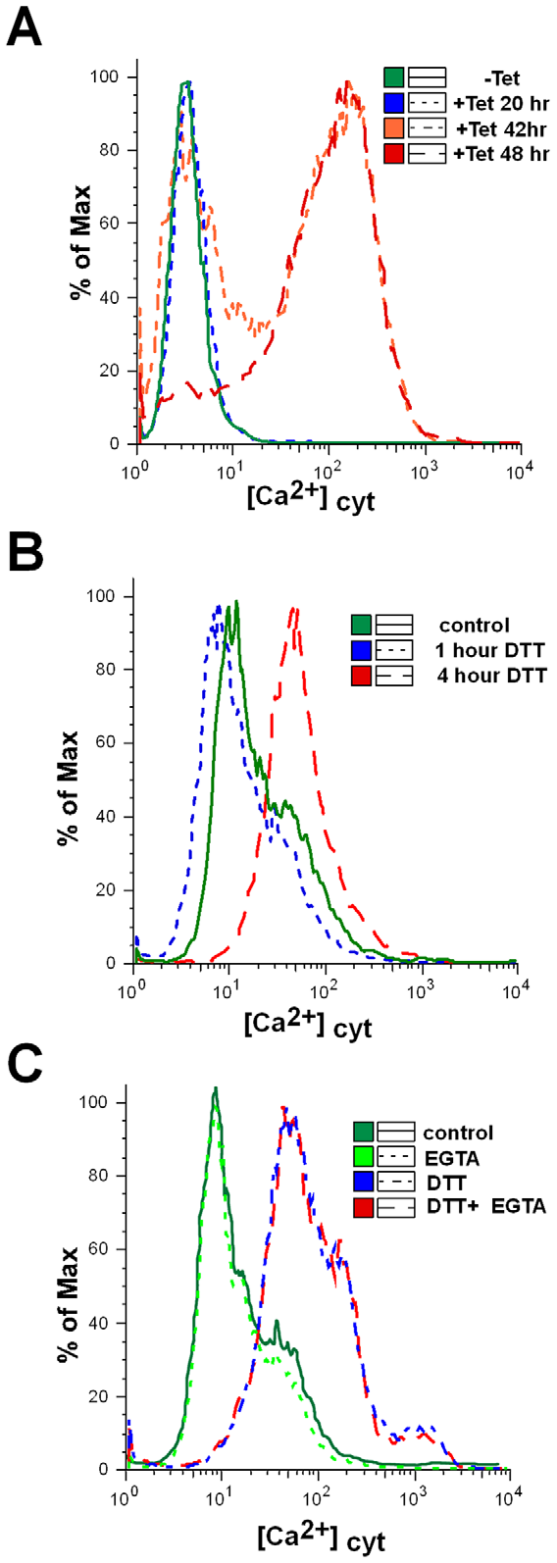
Cytoplasmic calcium concentration is elevated in SLS induced cells. **A.** Uninduced cells or SEC63 cells silenced for 20, 42 or 48 hours were treated with 1 µM Fluo-4-AM as described in Materials and Methods. The cells were then analyzed by FACS. **B.** Calcium increase upon DTT treatment. Cytoplasmic calcium was measured in cells treated with DTT for 0, 1 or 4 hours as described in Panel A. **C.** Calcium increase upon DTT treatment in calcium-free medium. Cytoplasmic calcium was measured in cells untreated or treated with DTT in medium lacking or containing 1 mM EGTA. Cells were treated with DTT for 4 hours. The symbols indicate the different treatments.

Recent studies have shown that an increase in cytoplasmic [Ca^2+^] occurs both at early and late stages of the apoptotic pathway. Ca^2+^ release from the ER induced by milder insults promotes cell death through apoptosis [51]. Trypanosomes, like other eukaryotes, maintain a low intracellular concentration of free Ca^2+^, and changes in the concentration of this ion were shown to affect the virulence of these organisms [52]. Several organelles were demonstrated to transport Ca^2+^ in an energy dependent manner, including the plasma membrane [53], endoplasmic reticulum [54], mitochondrion [55] and the acidocalcisome [56],[57]. In trypanosomes, the mitochondrion maintains a low resting level of Ca^2+^, but transiently accumulates large quantities of Ca^2+^ from the cytosol following Ca^2+^ influx across the plasma membrane or after release from the acidocalcisome [58]. Indeed, death in *T. brucei* was shown to be associated with changes in the ability of the mitochondrion to modulate Ca^2+^ levels [58]. To examine changes in cytoplasmic [Ca^2+^] during SLS, the level of cytoplasmic Ca^2+^ was examined using fluo-4-AM [59]. The results, shown in Figure 8-A, demonstrate an increase of cytoplasmic [Ca^2+^] mostly 42 to 48 hours following induction of SEC63 silencing. An increase in cytoplasmic [Ca^2+^] was also observed following treatment with DTT (Fig. 8-B). The results suggest that SLS is associated with perturbations of Ca^2+^ homoeostasis. Note that changes in the cytoplasmic [Ca^2+^] originate from internal pools, since these changes were unaffected by the presence of external EGTA (Fig. 8-C).

### Mitochondria depolarization and reactive oxygen species (ROS) production during SLS

**Figure 9:**
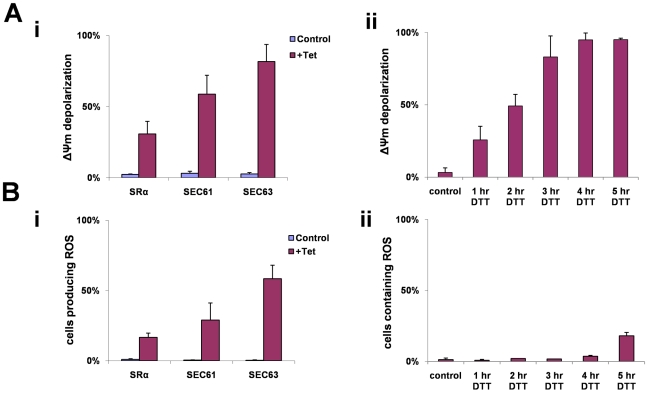
ΔΨ_m_ depolarization and ROS formation during SLS. **A.** Measurements of mitochondrial outer membrane potential. Cells were harvested and loaded with 150 nM Tetramethyl rhodamine methyl-ester (TMRM) in serum free medium. The samples were incubated in the dark for 15 min at 27°C and then analyzed by FACS. **i.** Cells uninduced (control) and silenced cell [72 hours (SRα) or 48 hours (SEC63, SEC61)] (+Tet) **ii.** Cells treated with 4 mM DTT for 0 to 5 hours. The data represent the percentage of cells lacking mitochondria membrane potential obtained from three independent experiments and the S.D. is indicated. **B.** Measurements of reactive oxygen species formation. Cells were harvested and incubated at 27°C for 30 min with 10 µM DCFH-DA. The cells were washed once with PBS and analyzed by FACS. **i.** Uninduced cells (control) and silenced cells [72 hours (SRα) or 48 hours (SEC63, SEC61)] (+Tet). **ii.** Cells treated with 4 mM DTT for 0 to 5 hours. The data represent the average of three independent experiments and the S.D is indicated.

A reduction in mitochondrial membrane potential (ΔΨm) has been observed during apoptosis. Tetramethyl rhodamine methyl-ester (TMRM) is a cationic lipophilic dye that enters cells and reversibly accumulates in the negatively charged mitochondrial matrix, depending on mitochondrial membrane potential [60]. Cells (uninduced) or after 3 days of silencing of SRα, SEC61 or SEC63 were incubated with TMRM, and the fluorescence of the dye was measured by FACS. The results (Fig. 9-Ai) indicate that up to 80% of the cells showed a decrease in membrane potential as a result of the silencing. To examine if membrane depolarization also takes place during DTT treatment, cells were treated with DTT for different time periods, and ΔΨm was measured. The results in Figure 9-Aii suggest that changes in ΔΨm were already observed after 1 hr, and after 3 hours, depolarization was observed in all cells.

During apoptosis, the inner mitochondrial membrane loses its integrity, and oxidative phosphorylation is uncoupled. When this occurs, oxidation of metabolites by O_2_ proceeds with electron flux not coupled to proton pumping, resulting in dissipation of the transmembrane proton gradient and ATP production, leading to the production of ROS. To measure ROS production that may result in part from the dissipation of the mitochondrial proton gradient, we used the sensitive probe 2′–7′ dichlorodihydrofluorescein (DCFH-DA). This non-fluorescent dye diffuses across cell membrane and is hydrolyzed to DCFH intracellularly. In the presence of ROS, DCFH is rapidly oxidized to highly fluorescent, dichlorofluorescein [61]. Indeed, ROS production was observed in SRα, SEC61 and SEC63 silenced cells (Fig. 9-Bi). ROS production was also monitored in DTT treated cells (Fig. 9-Bii). Interestingly, ROS production only appeared 5 hrs after DTT addition, although ΔΨm was already decreased after 3 hrs of DTT treatment (Fig. 9-Aii). This delay in observing ROS production after mitochondria permeabilization may reflect the different sensitivity of the assays used to measure ΔΨm and ROS. Note that DTT treatment could also affect ROS measurements because of its reducing capabilities.

One of the characteristic of changes involving the mitochondria during apoptosis is the leakage of proteins such as cytochrome C, which in metazoa, induces the activation of caspases [62]. Recently, endonuclease G (endoG) was described in trypanosomatid species [63]. This enzyme resides in the mitochondria and is released when cell death is triggered. The release of endoG from the mitochondria may represent a caspase-independent cell death mechanism. Since DNA fragmentation was observed in SLS-induced procyclic stage trypanosomes it was of great interest to examine if endoG is released in these cells. Indeed, endoG “leaked” to the cytosol in the SEC63 silenced cells (data not shown).

### TEM of SLS induced cells demonstrate morphological changes characteristic of apoptotic cells

**Figure 10:**
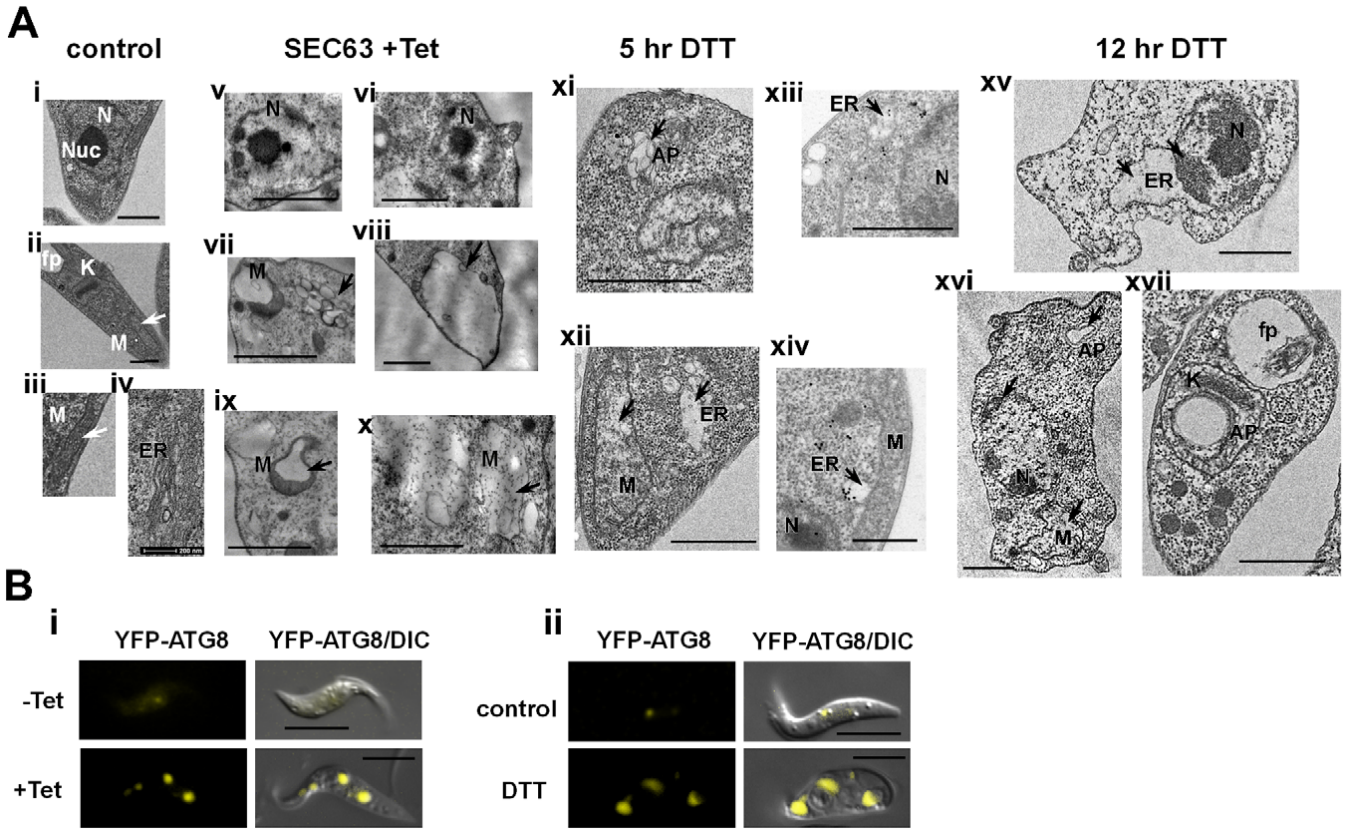
TEM of SLS-induced cells demonstrating morphological changes characteristic of apoptosis. **A.** Electron micrographs of untreated cells (**i-iv**), SEC63 cells after 48 hours of silencing (**v-x**), and cells treated with 4 mM DTT for 5 hours (**xi-xiv**) or 12 hours (**xv-xvii**); (**xiii-xiv**) sections were immunostained with anti BiP antibodies. N, nucleus; Nuc, nucleolus; M, mitochondria; ER, endoplasmic reticulum; AP, autophagosomes; fp, flagellar pocket. Scale bars: 1 µm. In each panel, the relevant structure is indicated with an arrow (as described in the text). **B.** Autophagosome formation under SEC63 silencing or DTT treatment. Cells carrying the silencing construct of SEC63 as well as the tagged YFP-ATG8 construct or parental cells carrying YFP-ATG8 constructs were visualized by live imaging using fluorescence microscopy. Parental cells were either **i.** uninduced (-Tet) or induced for 48 hrs (+Tet); **ii.** Untreated cells (control) or cells treated with DTT for 4 hrs (DTT), DIC- differential interference contrast. Scale bars, 5 µm.

Transmission electron microscopy (Fig. 10-A) of procyclic stage trypanosomes after 2 days of SEC63 silencing (v-x) or after 5 and 12 hrs of DTT treatment (xi-xiv, xv-xvii, respectively) show characteristic features of apoptotic cells. Untreated cells show nuclei with prominent central nucleolus (a) and equally distributed chromatin, mitochondria with no dilations (ii, iii), and a narrow ER (iv). In contrast, the SLS induced cells show condensed chromatin (v, vi, xv, xvi), expanded ER (xii, xiii, xiv, xv), and expanded, dilated mitochondria (vii, ix, x, xii, xvi). To verify that the expended organelle is indeed the ER, the procyclic cells slices were reacted with anti-BiP and visualized using gold-labeled antibodies by TEM. The results (xiii, xiv) demonstrate that the expanded compartment contained BiP and is therefore the ER lumen.

Note that chromatin condensation typical to apoptotic cells was not observed in SmD1 silenced cells, although these cells show major morphological perturbations such as accumulation of large vesicles in the cytoplasm (Figure S1-B). Autophagosomes appearing as membrane-surrounded bodies were observed in the cytosol (vii, xi, xvi), as well as inside the mitochondria (viii, xvii). Autophagy is induced as a survival response under starvation conditions [64], but autophagy was shown to regulate the endoplasmic reticulum during UPR [42],[65]. Autophagy involves the massive degradation of organelles such as mitochondria (mitophagy). Autophagy was also shown to induce PCD [66]. To examine if autophagy is induced during SLS, the formation of autophagosome was monitored using tagged ATG8. ATG8 is conserved from yeast to mammals and is commonly used as an autophagosome marker. ATG8 was tagged by *in situ* insertion of yellow fluorescent protein (YFP) to the authentic site both in parental cells and in cells expressing the SEC63 silencing construct. The results in Figure 10-B demonstrate that upon silencing of SEC63 (Fig. 10-Bi), or prolonged treatment with DTT (Fig. 10-Bii), clear autophagosome formation was observed. The most obvious change was the increase in autophagosomes number and size.

## Discussion

In this study, two major questions were addressed. We wished to determine whether trypanosomes are able to change their transcriptome as a result of ER stress like other eukaryotes, and whether this process is mediated or governed by the stress-induced mechanism, SLS [23]. Our results suggest that ER stress induces a very distinct response; the transcriptome is changed in a manner similar to changes observed in other eukaryotes during UPR. The changes in the transcriptome do not result from transcriptional programming as in other eukaryotes, but by stabilizing mRNAs that are needed to execute the ER stress response, resembling the heat-shock response in trypanosomes [19]. However, when ER stress is persistent, SLS is induced, suggesting that SLS does not replace the UPR but triggers programmed cell death under severe ER stress in both stages of the parasite. We therefore suggest that the biological role of SLS is to serve as the apoptotic branch of UPR and induce a very rapid and aggressive PCD response to eliminate unfit parasites from the population.

### Comparison of transcriptome during ER stress in trypanosomes to UPR in other eukaryotes

In this study, the changes of the transcriptome upon DTT treatment were compared to similar changes in yeast and metazoa. The data show that procyclic stage trypanosomes are able to change their transcriptome in response to ER stress. The most obvious change is the up-regulation of genes that are involved in protein secretory functions and the down-regulation of genes harboring signal-peptide and transmembrane domains. In yeast, the majority of the up-regulated genes play a role in secretion, phospholipid metabolism, protein glycosylation, vesicular transport, cell wall biosynthesis, vacuolar targeting and ER-associated degradation [3]. All together the UPR increased the level of proteins that lead to enlargement of the ER lumen, enhance export of the misfolded proteins for degradation [endoplasmic reticulum associated degradation (ERAD)] or enhance their transport from the ER via the vesicular transport. Very similar data was observed during UPR in *C. elegans*. [33]. In human cells, genes involved in targeting of proteins to the ER are induced, but most of these transcripts are only slightly induced, suggesting that modest changes in the level of their transcripts are sufficient for an adaptive response to ER stress [35]. In organisms such as *S. cerevisiae*, *D. melanogaster* and *C. elegans*, the specificity of the transcriptome changes during UPR was refined by examining the effect in IRE/XBP-1 mutants and their homologues [3],[33],[34]. In trypanosomes the specificity of the response to ER stress was refined only by kinetic analysis of the induced genes and therefore not all the affected genes might be directly linked to the ER stress response. However, the results indicate that in trypanosomes, the largest group of up-regulated genes, function in protein secretion and ER related functions similar to the proportion of such genes in yeast, fly, worm and human under UPR. Note, that as opposed to yeast, in trypanosomes, a large number of genes involved in signaling (17% vs. 8%) were up-regulated during the ER stress response, suggesting that signaling pathways are activated to execute this response. Of interest are also genes involved in movement, which resembles the signaling of chemotaxis in bacteria, enabling the microorganism to escape from chemical stressors by moving to a different microenvironment. Another important response of the UPR described in *D. melanogaster* suggest that IRE1 mediates the rapid degradation of a specific subset of mRNAs, encoding for genes harboring signal-peptide or transmembrane domains. This response helps to reduce the burden on the translocation and folding machinery [34]. Indeed, among the trypanosome genes that are down-regulated under ER stress, significant numbers contain signal-peptide or transmembrane domains.

### ER stress in trypanosomes

A recent study investigated the changes of *T. brucei* transcriptome during development as well as in response to tunicamycin or DTT treatments in bloodstream form trypanosomes [24]. This study reached the conclusion that while trypanosomes regulate mRNA abundance during the developmental cycle, they do not show significant changes in response to ER stress. The study failed to detect changes in BiP in response to ER stress and concluded that UPR does not exist in trypanosomes, primarily because these parasites live in homeostatic conditions, especially in the mammalian host. Our study reached an opposite conclusion. First, we observed major changes in the transcriptome, and, in addition, we observed changes in BiP expression during ER stress in both procyclic and bloodstream form trypanosomes. Moreover, we observed ER dilation at the same time at which we observed BiP up-regulation (1 and 5 hours) [(Fig. 3Bii, Fig. 10A-(xii, xiii, xiv)]. Note that the discrepancy between these studies can be easily reconciled given the fact that different DTT concentrations were used (1mM DTT vs. 4 mM DTT in our study). As noted in the previous study and as presented here, prolonged exposure to ER stress induces death. Here, we show that this death is associated with SLS during the entire life cycle of the parasite.

Another recent study on the enzyme UDP-glucose:glycoprotein glucosyltransferase (UGGT) demonstrated that null mutations of this gene in *T. brucei* bloodstream form do not induce BiP production. These results suggest that proteins involved in ER glycosylation are not tightly regulated in the trypanosome bloodstream form which might reflect the need for an extremely high flux of glycoprotein synthesis and export of VSG [67]. Interestingly, deletion of UGGT in *T. cruzi* has been reported to increase BiP expression, suggesting that in *T. cruzi* there is an ER stress response that senses the misfolded proteins and compensates for the lack of UGGT by up-regulating BiP [68]. All these data suggest that the response to ER stress may vary during the life cycle and among trypanosomatid species. However, the SLS induction in both procyclic and bloodstream from parasites as presented in this study, suggest that induction of ER stress by DTT or by major perturbation to the ER integrity and other cellular membranes, induce a systematic response. The response might be induced only under very severe stress that perturbs the membranes, since RNAi silencing of the post-translational pathway by depletion of SEC71 that does not harm membrane protein biogenesis did not induce SLS [46].

### How the response to ER stress is induced in trypanosomes, and how the mechanism is related to UPR in other eukaryotes

Conventional transcription regulation is absent in trypanosomes and therefore it was accepted that these organisms lack IRE1 and XBP1. In this study, we demonstrate a unique novel mechanism to induce UPR based on differential stabilization of mRNAs. The ER stress response resembles the heat shock regulation in trypanosomes, which is regulated mostly by mRNA stability and preferential translation [69]. mRNA stability is considered the major post-transcriptional mechanism that determines the trypanosome transcriptome [19]. The mechanism of the preferential stabilization under ER stress observed in this study is currently unknown. Stability of particular mRNAs can be regulated by two different mechanisms. RNA binding proteins can either stabilize the mRNAs under ER stress or a protein that normally destabilizes the mRNA can be inactivated during stress. Future challenges will be to identify the target site on the mRNAs that are the target of this mechanism, and further, to identify the cognate RNA binding protein(s) that mediates this regulation.

PERK is one of the key factors regulating UPR in metazoa. As opposed to yeast, which lack PERK, trypanosomes possess three potential PERK-homologues (TbITF2K1-K3) [70]. However, only one of these kinases, TbeIF2K2, carries a transmembrane domain and is able to phosphorylate eIF2α on Thr169, homologues to Ser51 of other eukaryotes [70]. This kinase level is reduced in high density culture but there is no evidence that this kinase participates in the ER stress response. Unexpectedly, the protein was localized to the flagellar pocket of the parasite [70]. No change in protein synthesis arrest as a result of DTT treatment was observed in cells silenced for this factor using RNAi (our unpublished results). Thus, TbeIF2K2 is most probably not involved in ER stress. Note that heat-shock in trypanosomes causes polysome collapse and translational shut-off independently of eIF2α phosphorylation, which takes place during the heat-shock response in other eukaryotes [69]. It remains possible that the other TbeIF2K kinases [70] function in ER stress. However, these kinases lack a transmembrane domain, and to function in the ER stress response these kinases must somehow associate with the ER membrane. Studies are in progress to decipher the possible role, if any, of these two kinases (TbeIF2K 1, 3) in ER stress and/or SLS.

UPR was shown to induce autophagy in yeast and mammals [65]. It was therefore interesting to observe autophagy in SLS-induced cells. The autophagy pathway functions as backup mechanism for ERAD, when substrates destined for degradation overwhelm the ERAD capacity [65]. This could also be the function of autophagy under ER stressors or silencing of SEC63 observed in this study.

### SLS with respect to other reported PCD inducers in trypanosomes

The presence of PCD in unicellular organisms and in trypanosomatids was initially viewed with skepticism [26]. The major argument against the presence of PCD in these organisms is the absence in trypanosomes of caspases that function in PCD. However, PCD was shown to be induced by prostaglandin D2 (PGD2), a molecule secreted primarily by the stumpy form of the parasite [71]. Trypanosomes control their density within the mammalian host by secreting a quorum sensing molecule (SIF) [72]. Slender parasites secrete SIF, which induces differentiation to the stumpy form. Stumpy parasites are pre-adapted for transmission to the fly but respond to PGD2 by PCD in the blood. Thus, the altruistic removal of stumpy forms assures persistent infection [71],[73]. The PCD associated with the prostaglandins involves an increase in ROS. It was suggested that trypanosomes might exhibit a primitive form of apoptosis that does not depend on proteases but instead depends on ROS formation [26]. This ROS signal can lead to stabilization of mRNAs encoding for proteases and nucleases that are needed to execute PCD. The death pathway executed by ER stress via SLS might be another altruistic pathway present in these parasites that was developed to remove the unfit parasites from the population under stress.

### Is SLS essential for PCD or is it stimulated to enhance PCD?

One can envision a mechanism by which ER stress induces imbalance of Ca^2+^ homeostasis. The increase in cytoplasmic [Ca^2+^] is most probably due to leakage from the malfunctioning ER, resulting from the reduced capacity of the ER to store Ca^2+^. This decreased capacity might stem from reduced levels of calreticulin and the ER-resident SERCA calcium pump, as well as the acidocalcisome Ca^2+^ transporters [74],[75]. These proteins either carry a signal-peptide or are polytopic membrane proteins. Indeed, major defects in biogenesis of both polytopic membrane proteins and signal peptide-containing proteins were observed in SEC63 and SEC61 silenced cells [46]. The ER stress induced by DTT and 2-DG may also affect the activity of the ER chaperone and the Ca^2+^ pumps.

In eukaryotes, Ca^2+^ from the ER or cytosol moves to the mitochondrial outer membrane through voltage dependent ion channel (VDAC) [76]. This leads to induced opening of the mitochondrial permeability transition pore (PTP) resulting in matrix swelling [51],[77]. Such changes cause the rupture of the outer membrane of the mitochondria, and release of apoptotic factors such as cytochrome C [78]. The rise in the mitochondrial Ca^2+^ stimulates the generation of ROS, and the opening of PTP causes dissipation of the ΔΨm, as was also observed in SLS-induced cells.

The PCD observed by silencing of ER functions (SRα, SEC61, SEC63) or by ER stressors can be explained by perturbations induced by Ca^2+^, leading to ROS production [79]. If so, what is the role of SLS and why is it required? We propose that SLS function is to speed up the death process. SLS is induced when response to ER stress fails to restore homeostasis, and it resembles apoptosis that takes place in mammalian cells under persistent ER stress [13],[80].

Very little is known about how the switch between protective and destructive pathways is regulated in eukaryotes. In metazoa a defined regulatory circuit that controls the switch from protective UPR to the pro-apoptotic pathway exists [81]. It was suggested that transient PERK signaling protects cells by temporarily dampening cellular protein synthesis and thus reducing the misfolded protein level in the ER. However, persistent PERK signaling could ultimately impair cell viability if extended translational inhibition interrupted the generation of proteins vital for cellular homeostasis [81]. An analogous situation may exist in trypanosomes. The severe inhibition of protein synthesis emerging from the drastic decrease in all mRNAs due to elimination of *trans*-splicing by SLS may lead to drastic reduction in the synthesis of proteins that are also required for the response to ER stress, and may resemble the physiological effects elicited by prolonged PERK induction in metazoa. SLS may accelerate cell death, rapidly eliminating unfit organisms from the population. One of the most intriguing and yet unresolved question is the nature of the signaling pathway that senses ER stress and transmits it to the nucleus to initiate the SLS response.

## Materials and Methods

### Cell growth and transfection

Procyclic forms of *T. brucei* strain 29–13 which carries integrated genes for T7 polymerase and the tetracycline repressor [82], were grown in SDM-79 [83] supplemented with 10% fetal calf serum in the presence of 50 µg/ml hygromycin B and 15 µg/ml G418.

The bloodstream form (BSFs) of *T. brucei* strain 427, cell line 1313–514 (a gift from C. Clayton, ZMBH, Heidelberg, Germany) [84] were aerobically cultivated at 37°C under 5% CO_2_ in HMI-9 medium [85] supplemented with 10% fetal calf serum containing 2 µg ml^−1^ G418 and 2.5 µg ml^−1^ phleomycin.

The oligonucleotides used in this study are listed in Table S1. Generation of the stem-loop silencing construct for SEC61α (Tb11.02.4100) using the oligonucleotides 104–106 was performed as previously described [86]. The construction of SEC63 and SRα silencing constructs was previously described [23],[46]. The construct to silence SEC63 in BSF was prepared by cloning a polymerase chain reaction (PCR) product generated using oligonucleotides 1111–1112 to the p2T7TA-177 vector. The transfection was performed as previously described [87]. Transgenic cell lines were selected on hygromycin B. To generate the YFP–ATG8 (Tb927.7.5910) fused protein, a PCR fragment was amplified using the oligonucleotides 829–830, covering positions 1 (first ATG) to the end of the protein. The vector, p2829 YFP-Dhh1, was digested with *Hind*III and *Not*I, and the fragment coding for ATG8 was cloned using the same sites [88]. The cloning creates an N-terminal fusion, and the *Hind*III site was used for fusing the gene and YFP. For integration, the plasmid was linearized with *Xcm*I at position 276 from the ATG. Cloned transgenic cell lines were selected on blasticidin.

### Microarray analysis

Total RNA was isolated from untreated cells and cells treated with DTT for 1 or 3 hours. RNA quality was determined using the Eukaryote Total RNA Nano 6000 assay kit (Agilent Technologies) on the Agilent Technologies 2100 Bioanalyzer. To generate the fluorescently labeled cRNA, total RNA was labeled using the Ambion Amino Allyl MessageAmp II aRNA kit (Ambion). A classical balanced block design with dye swap was used for comparing between RNA extracted following 1 hr DTT treatment, or 3 hr DTT treatment, and RNA extracted from uninduced cells. Arrays were blocked with 1% BSA, 0.1% SDS, 53 SSC at 42°C for 1 h, rinsed with double-distilled water, and air-dried. Next, 1.5 µg of cyanine 3-labeled and 1.5 µg of cyanine 5-labeled aRNA were fragmented and hybridized to the *T. brucei* DNA microarrays obtained through NIAID's Pathogen Functional Genomics Resource Center (managed and funded by the Division of Microbiology and Infectious Diseases, NIAID, NIH, DHHS, and operated by the J. Craig Venter Institute) using the Gene Expression hybridization kit (Agilent Technologies) for 16 h at 60°C in a hybridization oven. After hybridization, each array was subjected to three washes: first wash with 2× SSC, 0.1% SDS for 1 min at 55°C, and then 0.1× SSC, 0.1% SDS for 1 min, and finally with 0.1× SSC for 1 min, at room temperature. Arrays were scanned using the dual laser scanner (Agilent G2505B Microarray scanner). The data were then extracted from images using Spotreader software (Niles Scientific). Flawed features identified by visual inspection of the array images were flagged, and the data from these features were removed prior to further analysis.

### Northern blot analysis

Total RNA was prepared with Trizol reagent and 20 µg/lane was separated on a 1.2% agarose gel, containing 2.2 M formaldehyde. Small RNAs were fractionated on a 6% (w/v) polyacrylamide gel containing 7M urea. The RNA blots were hybridized to oligonucleotides or [α-^32^P]-random labeled probes (Random Primer DNA Labeling Mix, Biological Industries, Co.). The oligonucleotides used to prepare the probes are detailed in Table S1.

### mRNA stability analysis

Untreated cells and cells after 1.5 hour of DTT treatment (10^7^ cells/ml) were divided into 50 ml aliquots for each time point, concentrated and resuspended in 5 ml of the remaining growth medium. The concentrated cells were incubated for 30 min at 27°C, treated with 2 µg/ml sinefungin (Sigma) for 10 min, and then 30 µg/ml of actinomycin D were added (Sigma). Aliquots were taken at different time points (0–90 min). For each time point, total RNA was prepared and the samples were subjected to Northern analysis. Quantization by densitometry was performed using ImageJ software, and the results were used for calculating the half-life of the mRNAs.

### Western blot analysis

Whole cell lysates (10^6^ cell equivalents per lane) were fractionated by SDS-PAGE, transferred to PROTRAN membranes (Whatman), and probed with anti BiP (diluted 1∶5000), kindly provided by Prof. James Bangs (University of Wisconsin-Madison, Madison, USA); anti tSNAP42 antisera (diluted 1∶7500) kindly provided by Prof. Vivian Bellofatto (UMDNJ- NJ, USA); and anti p58 antibodies raised by our group against a cytoplasmic protein Tb11.46.0009 (1∶4000). The bound antibodies were detected with goat anti-rabbit immunoglobulin G (IgG) or anti-mouse IgG coupled to horseradish peroxidase, and were visualized by ECL (Amersham Biosciences).

### Immunofluorescence assay

Cells were washed with PBS, mounted on poly-L-lysine-coated slides, fixed in 4% formaldehyde, and immunofluorescence was performed as described in [23] using anti tSNAP42 antisera. The cells were visualized with a Zeiss LSM 510 META inverted microscope.

### Propidium Iodide (PI) staining for FACS analysis

*T. brucei* cells, silenced for various genes, were fixed in 70% Ethanol-30% PBS and stored at 4°C overnight. Cells were then washed once with PBS and incubated on ice for 30 minutes to enable rehydration. The samples were resuspended in PBS containing 50 µg/ml RNase A (Roche Diagnostics) for 30 minutes at 4°C and stained with 50 µg/ml Propidium Iodide (Sigma). Samples were analyzed by FACS using the FACStar plus (Becton Dickinson, San Jose, CA) and the CellQuest list mode analysis software.

### Measurement of mitochondrial membrane potential

*T. brucei* cells following the different treatments were harvested and loaded with 150 nM TMRM (Invitrogen) in serum-free medium. The samples were incubated in the dark for 15 min at 27°C and then analyzed by FACS.

### Detection of intracellular reactive oxygen species

Reactive oxygen species (ROS) were measured by flow cytometry using DCFH-DA (Sigma). Following the different treatments cells were harvested and incubated at 27°C for 30 min with 10 µM DCFH-DA. The cells were then washed once with PBS and analyzed by FACS using the CellQuest list mode analysis software.

### Measurement of intracellular calcium

For calcium measurements, 10^6^ cells were loaded with 1 µM Fluo-4-AM (Invitrogen) for 1 hour at 27°C. The cells were washed three times with PBS and resuspended in PBS. Staining was evaluated by FACS. Results were analyzed using FlowJo software.

### DNA laddering assay

Cells were pelleted by centrifugation, washed with PBS, resuspended in extraction buffer (10 mMTris pH 8.0, 0.1 mM EDTA pH 8.0, 20 µg/ml RNase, 0.5% SDS), incubated at 37°C for 1 hour, and for an additional 3 hours at 50°C in the presence of 4 µg/ml proteinase K. After phenol:chloroform (1∶1 V/V) and chloroform extractions, and ethanol precipitation, the DNA was treated with 10 µg/ml RNaseA for 1 hour at 37°C and 10 µg was electroporated on 1.8% Agarose gel containing 0.5 µg/ml ethidium bromide and visualized under UV light.

### TUNEL assay

Detection of cleavage of genomic DNA was performed by using the In-Situ Cell Death Detection Kit, FITC (Roche Molecular Biochemicals) according to manufacturer's instructions.

### Phosphatidylserine exposure assay

Trypanosomes were reacted with fluorescein isothiocyanate-labeled Annexin V antibodies (MBL^©^) and stained with propidium iodide according to the manufacturer's instructions. The cells were analyzed by FACS or visualized under a Zeiss LSM 510 META inverted microscope.

### Transmission electron microscopy

Trypanosomes were fixed with Karnovsky (4% paraformaldehyde, 2.5% glutaraldehyde) solution for 1 h at room temperature, and then at 4°C over night. Thereafter, the cells were incubated with 1% OsO_4_ for 1 h at 4°C. Samples were dehydrated in ethanol and embedded in Epon-812 according to standard procedures. Ultrathin sections were prepared using a LKB Ultratome III, stained with uranyl acetate and contrasted with lead citrate. The samples were visualized using a transmission electron microscope FEI Tecnai 120 kV.

### Immuno-electron microscopy

Trypanosomes were fixed with Karnovsky (2% paraformaldehyde, 0.5% glutaraldehyde) solution for 1 h at room temperature, and then at 4°C over night. Thereafter, the cells were incubated with 1% OsO_4_ for 1 h at 4°C. Samples were dehydrated in ethanol and embedded in Epon-812 according to standard procedures. Ultrathin sections were prepared using a LKB Ultratome III. The sections were floated in blocking buffer (10mM Tris pH 8.5, 0.05% Tween-20, 5% non-fat dry milk) for 30 minutes and then incubated with rabbit anti BiP antibodies, kindly provided by Prof. James Bangs (University of Wisconsin-Madison, Madison, USA) diluted (1∶20) in the blocking buffer (4°C, O.N) washed with wash buffer (10 mM Tris pH 8.5, 0.05% Tween 20) and incubated with Donkey anti rabbit IgG (H+L) conjugated to 18 nm gold particles (Jackson immune-research cat.# 711-215-152, diluted 1∶100) and washed again with wash buffer. The grids were stained with uranyl acetate. The samples were visualized using a transmission electron microscope FEI Tecnai 120 kV.

## Supporting Information

Figure S1 The PCD hallmarks chromatin condensation and PS exposure are not found in cells dying from SmD1 and PTB2 silencing. A. AnnexinV is not exposed on cells silenced for SmD1 and PTB2. Uninduced cells (-Tet) or SmD1 cells induced for 2 days (SmD1 +Tet 2 days) and PTB2 cells induced for 3 days (PTB2 +Tet 3 days), were reacted with fluorescein isothiocyanate-labeled AnnexinV antibodies (MBL^©^) and stained with propidium iodide according to the manufacturer's instructions. The cells were analyzed by FACS. B. Electron micrographs of SmD1 cells silenced for 2 days (i-iii). N, nucleus; Nuc, nucleolus; Scale bars, 1 mm.
(1.60 MB TIF)

Figure S2 Silencing of SEC63 in bloodstream form trypanosomes. Northern blot analysis of SEC63 RNAs. RNA was prepared from uninduced (-Tet) and silenced cells 2 or 3 days after induction (+Tet) and was subjected to Northern analysis with radio-labeled probes. The transcripts examined are indicated.
(0.38 MB TIF)

Table S1 The following oligonucleotides were used for detection of Northern blots or for plasmid construction
(0.09 MB PDF)

Table S2 Up-regulated genes of *T. brucei* under DTT treatment. The GenDB numbers, annotation and fold change of genes that were up-regulated under DTT treatment for 1 hour and 3 hours, obtained from the array analysis as described in Materials and Methods, are listed. The genes were categorized according to their biological function.
(0.08 MB XLS)

Table S3 UPR up-regulated genes from *S. cerevisiae*; *C. elegans*; *D. melanogaster* and *H. sapiens*. To sieve genes that participate in UPR in *S. cerevisiae*, we repeated the computational procedure as described in the original article [3]. Genes participating in UPR in *C. elegans* were derived from the supplementary tables S1 and S2 of the article [32]. In *D. melanogaster*, fold changes were averaged over all replicates under each experimental condition. Up-regulated genes participate in UPR were identified as genes whose level increased by 1.5 fold following DTT treatment and whose fold change was at least twice as that of the fold change in the mutants (Ire1- and Xbp1-) [33]. In *H. sapiens*, genes were identified as UPR candidates if their level following DTT treatment for 1 hour was increased by at least 1.5 fold (versus untreated), and if their fold change after 4 hours was greater than after 1 hour of treatment [34].
(0.18 MB XLS)
