# Supplementary figures and images for: Persistent ER Stress Induces the Spliced Leader RNA Silencing Pathway (SLS), Leading to Programmed Cell Death in Trypanosoma brucei
Source: PLoS Pathog. 2010 Jan 22;6(1):e1000731. doi: 10.1371/journal.ppat.1000731 (PMC2809764; doi:10.1371/journal.ppat.1000731)

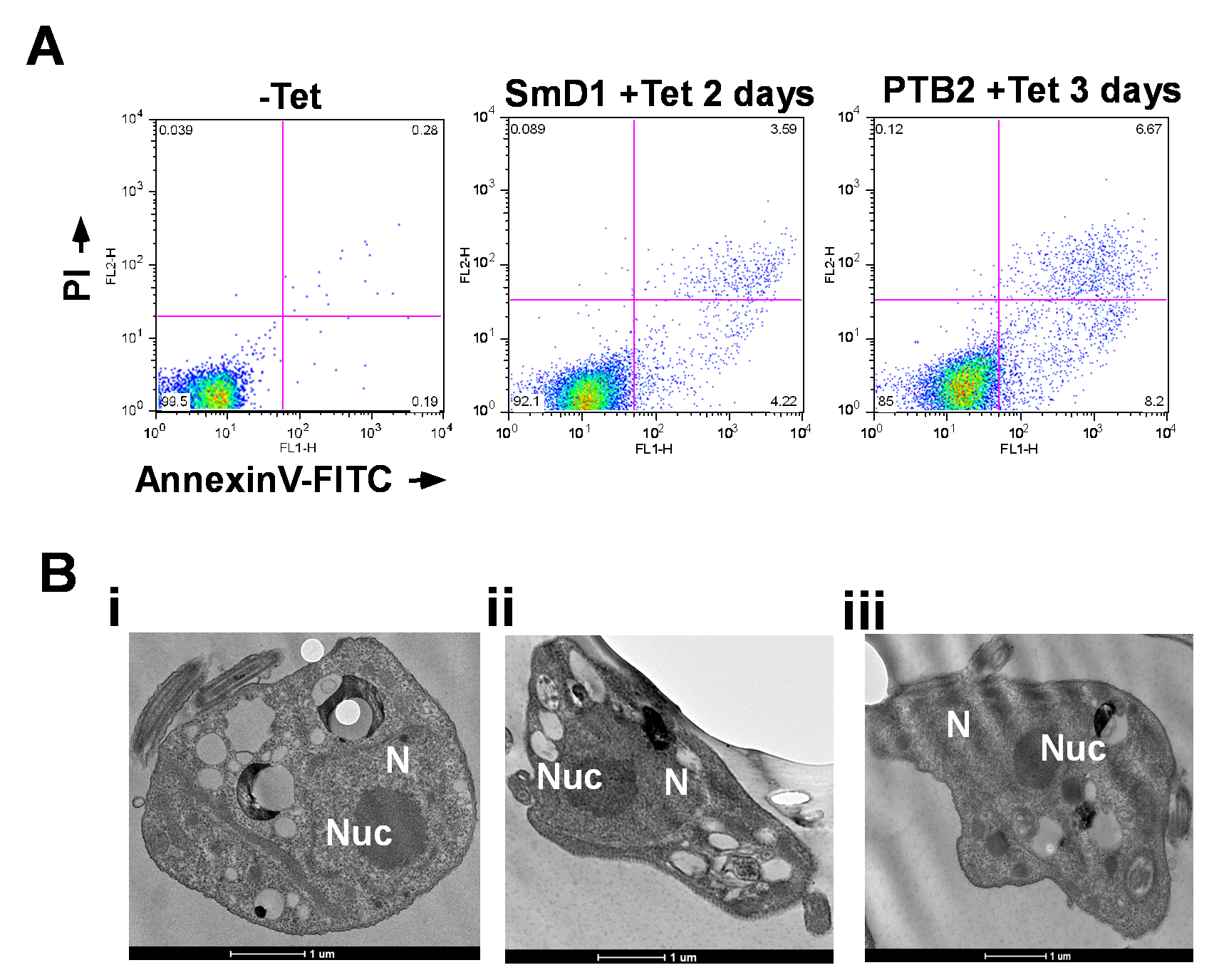

Supplement: Figure S1 — The PCD hallmarks chromatin condensation and PS exposure are not found in cells dying from SmD1 and PTB2 silencing. A. AnnexinV is not exposed on cells silenced for SmD1 and PTB2. Uninduced cells (-Tet) or SmD1 cells induced for 2 days (SmD1 +Tet 2 days) and PTB2 cells induced for 3 days (PTB2 +Tet 3 days), were reacted with fluorescein isothiocyanate-labeled AnnexinV antibodies (MBL©) and stained with propidium iodide according to the manufacturer's instructions. The cells were analyzed by FACS. B. Electron micrographs of SmD1 cells silenced for 2 days (i-iii). N, nucleus; Nuc, nucleolus; Scale bars, 1 mm. (1.60 MB TIF) [file ppat.1000731.s001.tif]

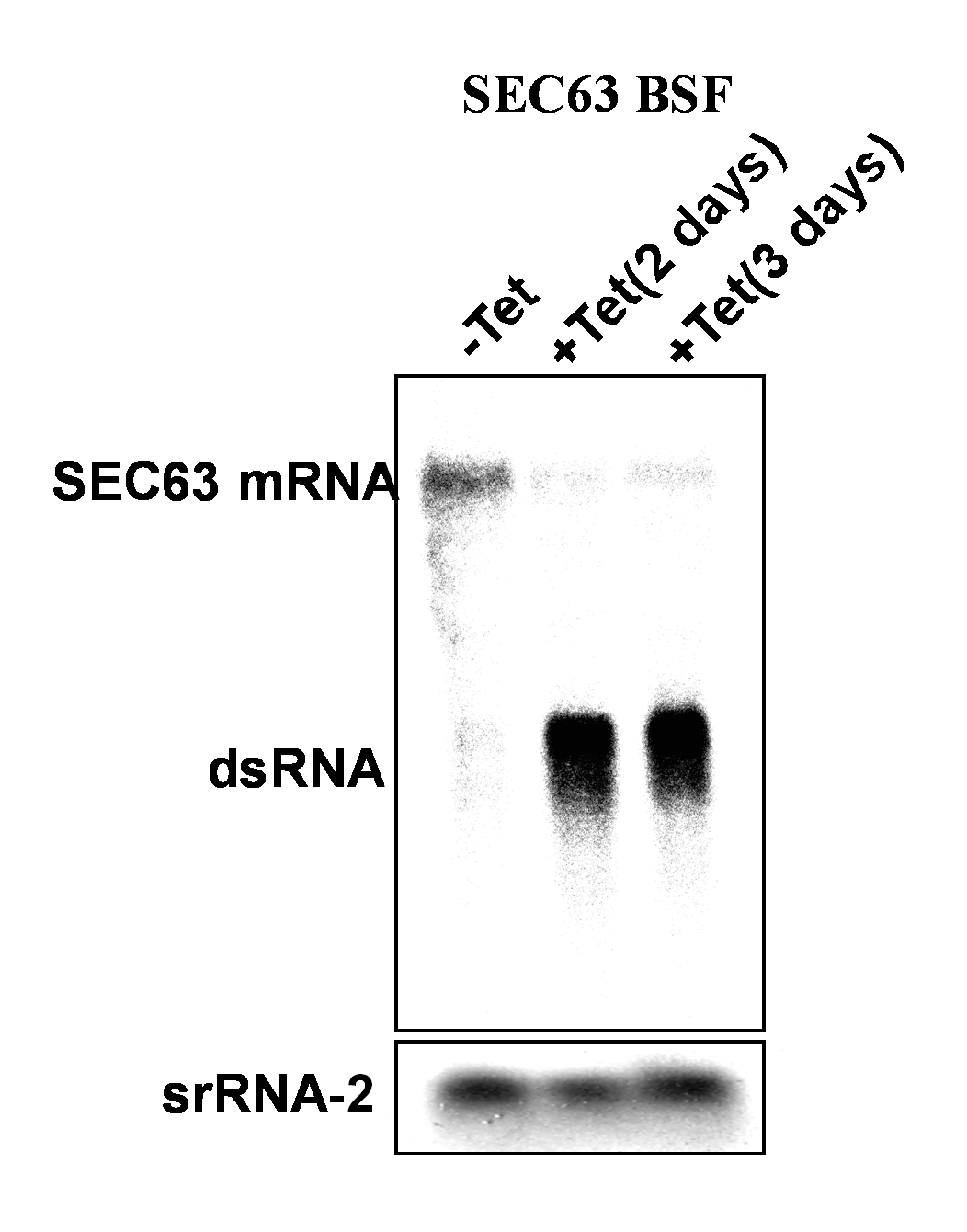

Supplement: Figure S2 — Silencing of SEC63 in bloodstream form trypanosomes. Northern blot analysis of SEC63 RNAs. RNA was prepared from uninduced (-Tet) and silenced cells 2 or 3 days after induction (+Tet) and was subjected to Northern analysis with radio-labeled probes. The transcripts examined are indicated. (0.38 MB TIF) [file ppat.1000731.s002.tif]
