# Supplementary material for: Persistent ER Stress Induces the Spliced Leader RNA Silencing Pathway (SLS), Leading to Programmed Cell Death in Trypanosoma brucei
Source: PLoS Pathog. 2010 Jan 22;6(1):e1000731. doi: 10.1371/journal.ppat.1000731 (PMC2809764; doi:10.1371/journal.ppat.1000731)
Supplement: Table S1 — The following oligonucleotides were used for detection of Northern blots or for plasmid construction (0.09 MB PDF) [file ppat.1000731.s003.pdf]

S-1

Table S-1: Oligonucleotides

**The following oligonucleotides were used for detection of Northern blots**			
**162920**	5'-TCTCCTCACCCCCTCGCG	sense	complementary to positions 123-141 of tubulin
**162921**	5'-ATGCAGATAGCCTCACGC	antisense	complementary to positions 3-20 of tubulin
**TBNT8.1 Sense**	5'- CGCAACATGCATGCA TCCTTCTTG	Sense	Complementary for positions 42-66 of TbNT8.1 -Tb11.02.1100 coding region
**TBNT8.1 anti**	5'- TCGATGCCTTGATGA TGCAC	antisense	Complementary for positions 522-541 of TbNT8.1 -Tb11.02.1100 coding region
**8977**	5′-ACTAACGCTATTATTAG AACAGTTTCTGTACTAT	Sense	specific for *T. brucei* SL RNA 5′ from positions 1 to 35
**0244C02**	5′-TTAATACGACTCAC TATAGGGAGAAAAAA AATAAAAAAAATA	Sense	complementary to positions 143 to 161 with respect to the +1 position of the SL RNA transcript bearing the T7 promoter (underlined)
**srRNA-1**	5`-TAATGCGCCGAACTCACA AC	Antisense	complementary to the small ribosomal RNA -1 from position 177 to 196
**srRNA-2**	5`-GGC TTA GAG GCG TTC AGC CGA-3`	Antisense	complementary to the s small ribosomal rRNA-2 from position 134 to 154
**953610**	5`- CCGCTCGAGAGCCGGAGC TTGCTCTG	sense	Complementary for positions 1-16 of 7SL RNA including *Xho*I site (underlined)
**953611**	5`-CCCAAGCTTCCGCCTCGC GACGACACT	antisense	Complementary for positions 278-253 of 7SL RNA including *Hind*III site (underlined)
**1106**	5`- ATACGGGAAGCTATACAGG	sense	complementary to positions 73-92 of syntaxin Tb10.70.6010
**1107**	5`- TATTACGTCCTGCTGTGC	antisense	complementary to positions 426-444 of syntaxin Tb10.70.6010
**1098**	5`-GCACAAATGATGCTTTAC	sense	complementary to positions 136-154 of chaperone protein DnaJ Tb10.389.1150
**1099**	5`-CATATTACCCTCCTCACC	antisense	complementary to positions 417-435 of chaperone protein DnaJ Tb10.389.1150
**1102**	5`- GTGGAACAGTTCAGAAAC	sense	complementary to positions 24-43 of thioredoxin Tb09.160.2020
**1103**	5`-AGCTCCAATCACATGT CC	antisense	complementary to positions 251-273 of thioredoxin Tb09.160.2020
**1104**	5`-GTTCATCCTGGAGTTTG	sense	complementary to positions 986-1004 of protein disulfide isomerase Tb10.6k15.2290
**1105**	5`- ACATTAGCTGGTTTCTCAC	antisense	complementary to positions 1378-1397 of protein disulfide isomerase Tb10.6k15.2290
**The following oligonucleotides were used for plasmids construction**			
**104**	5`- CTAGTCTAGAGTCTGGCAT GTACGGTGACG	sense	from position 399 of the *SEC61* coding region, including a *Xba*I site (underlined); was used for PCR amplification of 400 bp for cloning into the pJM326 and pLew100 vectors, respectively, to generate the stem-loop construct

**105**	5`-CCCAAGCTTCATAAGAGGGACGCGGAAAC	antisense	from positions 798 of the *SEC61* coding region, including a *Hin*dIII site; was used for PCR amplification of 400 bp for cloning into the pJM326 vector to generate the stem-loop construct
**106**	5`-CGACGCGTCATAAGAGGGACGCGGAAAC	antisense	from positions 798 of the *SEC61* coding region, including a *Mlu*I site; was used for PCR amplification of 400 bp for cloning into the pLew100 vector to generate the stem-loop construct
**829**	5`-CCCAAGCTTCCATGAGTAAAAAAGATAGC	sense	from positions 1 of the *ATG8* coding region
**830**	5`-CCCGCGGCCGCTTAGCATCCAAATGTCG	antisense	from positions 363 of the *ATG8* coding region
**1111**	5`-CCCCTCGAGCTGAGGAGGCGCGTTTGA	sense	from position 502-520 of the *SEC63* coding region, including a *Xho*I site (underlined); was used for PCR amplification of 516 bp for cloning into the p2T7TA-177 vector [B. Wickstead, K. Ersfeld and K. Gull,. Mol. Biochem. Parasitol. **125** (2002), pp. 211–216], to generate silencing construct for transfection into BSF 1313-514 cell line**.**
**1112**	5`-CCCGGATCCGGAGAACTTCGATGTCGC	antisense	from positions 1000-1018 of the *SEC63*coding region, including a *BamH*I site; was used for PCR amplification of 516 bp for cloning into the p2T7TA-177 vector to generate silencing construct for transfection into BSF 1313-514 cell line**.**
